# Supplementary material for: SARS-CoV-2 spike protein variant binding affinity to an angiotensin-converting enzyme 2 fusion glycoproteins
Source: PLoS One. 2022 Dec 6;17(12):e0278294. doi: 10.1371/journal.pone.0278294 (PMC9725131; doi:10.1371/journal.pone.0278294)
Supplement: S1 File — (PDF) [file pone.0278294.s001.pdf]

Raw images and quantitative data.

Fig 1a: Full immunoblots

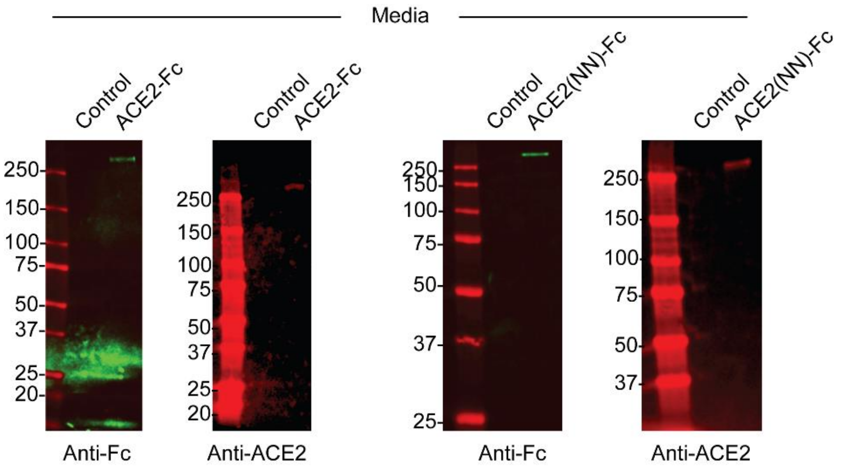

Fig 1b-d: Mean values with standard deviation

Viable Cell Density (x10^6)	**ACE2-Fc**										
	**Batch**	**Unfed 1**	**Unfed 2**	**Unfed 3**	**Unfed all**		**Fed 1**	**Fed 2**	**Fed 3**	**Fed all**	
	**Time**	**AVE. (n=3)**	**AVE. (n=3)**	**AVE. (n=3)**	**AVE.**	**S.D.**	**AVE. (n=3)**	**AVE. (n=3)**	**AVE. (n=3)**	**AVE.**	**S.D.**
	**Day 0**	0.30	0.30	0.30	0.30	0.00	0.30	0.30	0.30	0.30	0.00
	**Day 3**	3.45	3.77	3.77	3.66	0.18	3.30	4.09	3.83	3.74	0.40
	**Day 5**	8.40	9.18	9.18	8.92	0.45	10.13	11.21	8.61	9.98	1.31
	**Day 7**	8.85	9.94	9.94	9.58	0.63	9.41	10.74	10.00	10.05	0.67
	**ACE2(NN)-Fc**										
	**Batch**	**Unfed 1**	**Unfed 2**	**Unfed 3**	**Unfed all**		**Fed 1**	**Fed 2**	**Fed 3**	**Fed all**	
	**Time**	**AVE. (n=3)**	**AVE. (n=3)**	**AVE. (n=3)**	**AVE.**	**S.D.**	**AVE. (n=3)**	**AVE. (n=3)**	**AVE. (n=3)**	**AVE.**	**S.D.**
	**Day 0**	0.30	0.30	0.30	0.30	0.00	0.30	0.30	0.30	0.30	0.00
	**Day 3**	4.72	4.18	6.10	5.00	0.99	3.99	3.91	3.87	3.92	0.06
	**Day 5**	6.46	7.09	6.46	6.67	0.37	6.99	6.71	6.99	6.90	0.16
	**Day 7**	5.51	6.63	7.44	6.53	0.97	6.92	7.25	9.17	7.78	1.21
Viability	**ACE2-Fc**										
	**Batch**	**Unfed 1**	**Unfed 2**	**Unfed 3**	**Unfed all**		**Fed 1**	**Fed 2**	**Fed 3**	**Fed all**	
	**Time**	**AVE. (n=3)**	**AVE. (n=3)**	**AVE. (n=3)**	**AVE.**	**S.D.**	**AVE. (n=3)**	**AVE. (n=3)**	**AVE. (n=3)**	**AVE.**	**S.D.**
	**Day 3**	99.00	98.00	99.00	98.67	0.58	98.67	98.00	99.00	98.56	0.51
	**Day 5**	99.33	98.67	99.00	99.00	0.33	99.00	98.67	99.33	99.00	0.33
	**Day 7**	99.00	95.67	96.67	97.11	1.71	98.33	95.67	97.67	97.22	1.39

ACE2(NN)-Fc										
Batch	Unfed 1	Unfed 2	Unfed 3	Unfed all		Fed 1	Fed 2	Fed 3	Fed all	
Time	AVE. (n=3)	AVE. (n=3)	AVE. (n=3)	AVE.	S.D.	AVE. (n=3)	AVE. (n=3)	AVE. (n=3)	AVE.	S.D.
Day 3	98.00	98.00	97.67	97.89	0.19	98.33	98.33	98.33	98.33	0.00
Day 5	96.67	94.67	96.67	96.00	1.15	97.67	96.33	97.67	97.22	0.77
Day 7	84.33	87.33	89.00	86.89	2.36	91.67	92.00	94.00	92.56	1.26

Production (mg/L)

ACE2-Fc										
Batch	Unfed 1	Unfed 2	Unfed 3	Unfed all		Fed 1	Fed 2	Fed 3	Fed all	
Time	AVE. (n=3)	AVE. (n=3)	AVE. (n=3)	AVE.	S.D.	AVE. (n=3)	AVE. (n=3)	AVE. (n=3)	AVE.	S.D.
Day 3	0.12	0.36	0.28	0.25	0.12	0.15	0.22	0.12	0.16	0.05
Day 5	0.57	0.52	0.46	0.52	0.06	0.59	0.51	0.41	0.50	0.09
Day 7	0.65	0.83	0.59	0.69	0.13	0.68	0.66	0.58	0.64	0.05

ACE2(NN)-Fc										
Batch	Unfed 1	Unfed 2	Unfed 3	Unfed all		Fed 1	Fed 2	Fed 3	Fed all	
Time	AVE. (n=3)	AVE. (n=3)	AVE. (n=3)	AVE.	S.D.	AVE. (n=3)	AVE. (n=3)	AVE. (n=3)	AVE.	S.D.
Day 3	0.38	0.30	0.33	0.34	0.04	0.36	0.33	0.29	0.33	0.04
Day 5	0.72	0.61	0.58	0.64	0.08	0.81	0.68	0.61	0.70	0.10
Day 7	0.94	0.78	0.71	0.81	0.12	0.96	0.83	0.74	0.84	0.11

AVE.= Average, S.D. = Standard Deviation

Fig 1e: Full immunoblots

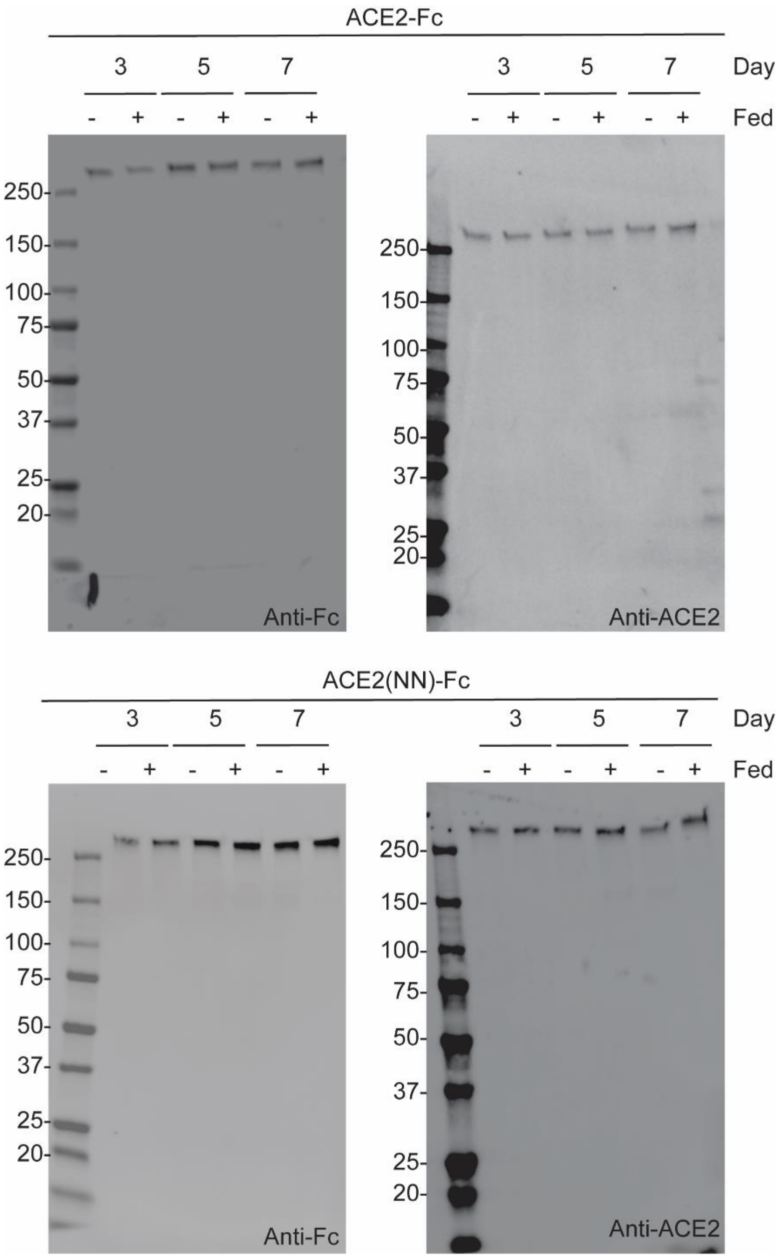

Fig 2b: Full immunoblots

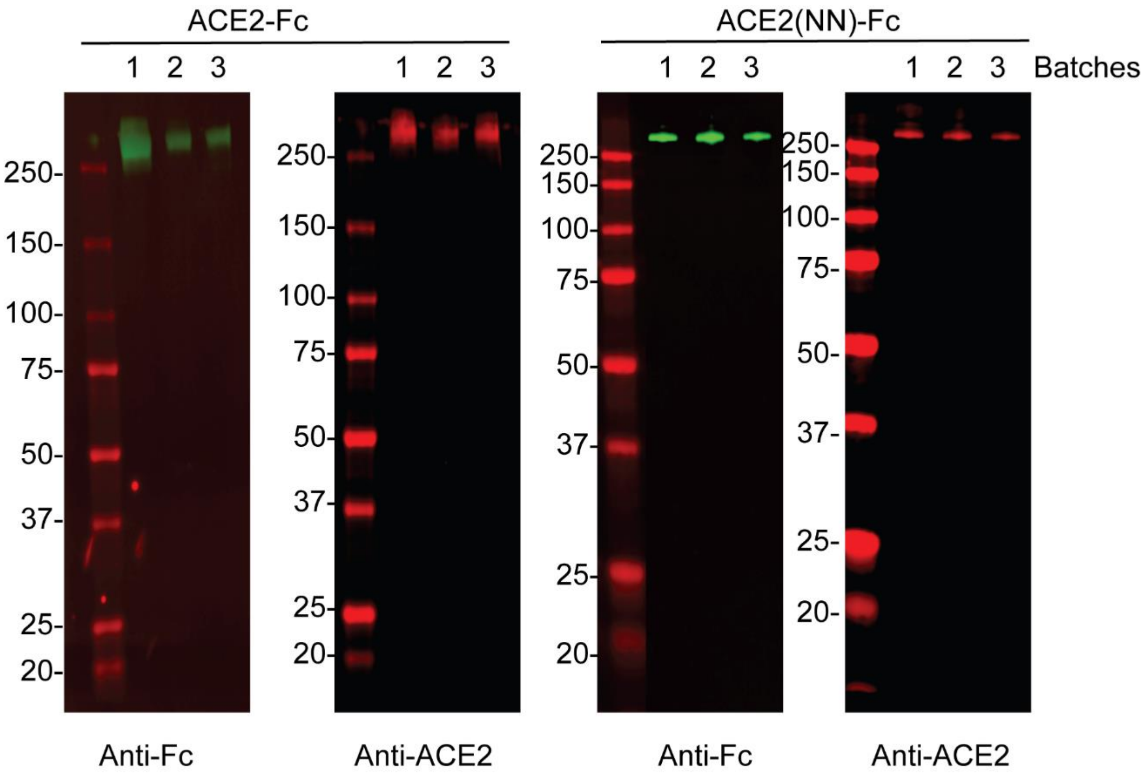

Fig 2c: Mean values with standard deviation

ACE2 Activity (mU/mg)										
**Batches**	**1**				**2**				**All**	
**Replicates**	n=1	n=2	n=3	n=4	n=1	n=2	n=3	n=4	AVE.	S.D.
**ACE2-Fc**	1606.72	1613.11	1643.79	1724.35	1227.73	1227.16	1328.88	1374.70	1468.30	200.25
**ACE2(NN)-Fc**	-2.94	-1.14	-4.28	-6.00	5.52	3.52	-3.11	5.60	-0.35	4.59

AVE.= Average, S.D. = Standard Deviation

Fig 3a: Full Coomassie-stained gels

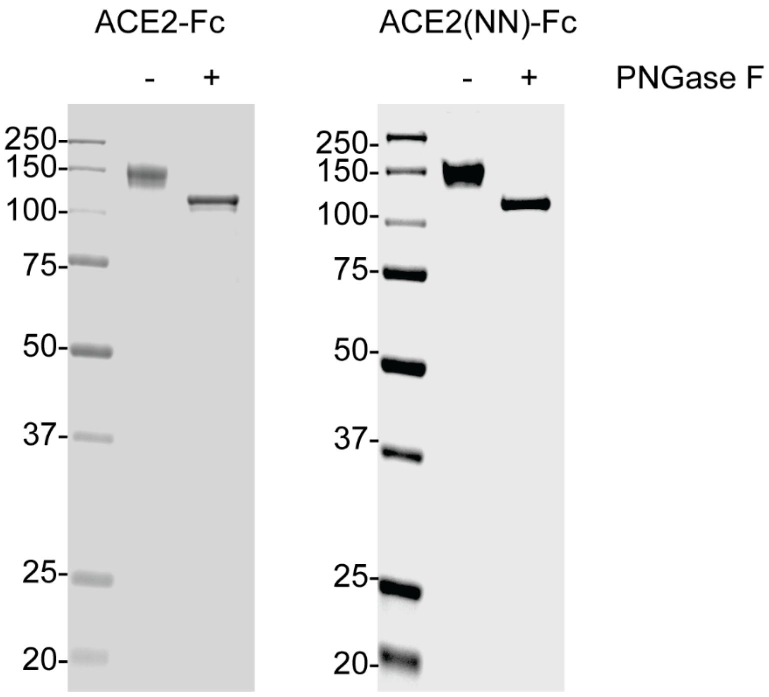

Fig 4c and 4d: Mean values with standard deviation

	T onset		Tm	
	AVE.	S.D.	AVE.	S.D.
ACE2-Fc	40.70	4.33	48.13	0.25
ACE2(NN)-Fc	42.71	2.89	57.27	6.59

AVE. = AVERAGE, S.D. = Standard Deviation
