# Supplementary material for: SARS-CoV-2 spike protein variant binding affinity to an angiotensin-converting enzyme 2 fusion glycoproteins
Source: PLoS One. 2022 Dec 6;17(12):e0278294. doi: 10.1371/journal.pone.0278294 (PMC9725131; doi:10.1371/journal.pone.0278294)
Supplement: S2 File — (PDF) [file pone.0278294.s002.pdf]

Supplemental tables

S1 Table: One- and Two-Way ANOVA results describing the degrees of freedom and p values.

One Way ANOVA			
Test	Figure	F (DFn, DFd)	*p* value
ACE2 Activity	Figure 2c	F (1, 12) = 62.69	P < 0.0001
Variant binding affinity to ACE2-Fc	Figure 5a	F (2, 23) = 17.92	P < 0.0001
Variant dissociation with ACE2-Fc	Figure 5b	F (2, 23) = 12.54	P = 0.0002
Variant binding affinity to ACE2(NN)-Fc	Figure 5d	F (2, 24) = 10.83	P = 0.0004
Variant dissociation with ACE2(NN)-Fc	Figure 5e	F (2, 23) = 6.319	P = 0.0065

Two Tailed ANOVA (Repeated measure - Time)				
Test	Figure	Factors	F (DFn, DFd)	*p* value
Cell Density of CHO cells expressing ACE2-Fc	Figure 1b	Interaction	F (3, 48) = 1.962	0.1322
		Day	F (3, 48) = 710.8	< 0.0001
		Fed	F (1, 16) = 2.886	0.1087
Cell Density of CHO cells expressing ACE2(NN)-Fc	Figure 1b	Interaction	F (3, 48) = 5.420	0.0027
		Day	F (3, 48) = 236.1	< 0.0001
		Fed	F (1, 16) = 0.2700	0.6104
Cell Viability of CHO cells expressing ACE2(NN)-Fc	Figure 1c	Interaction	F (2, 32) = 11.74	0.0002
		Day	F (2, 32) = 118.3	< 0.0001
		Fed	F (1, 16) = 29.23	< 0.0001
N-Glycan comparison	Figure 3d	Interaction	F (23, 48) = 3.454	0.0001
		Glycan Structure	F (23, 48) = 69.04	< 0.0001
		ACE2 Fusion Protein	F (1, 48) = 8.437e-007	0.9993
Spike variant binding affinity to ACE2-Fc and ACE2(NN)-Fc	Figure 5c	Interaction	F (4, 79) = 0.2420	0.9137
		Spike Variant	F (4, 79) = 13.63	< 0.0001
		ACE2 Variant	F (1, 79) = 0.1211	0.7287
Spike variant association rate to ACE2-Fc and ACE2(NN)-Fc	Figure 5d	Interaction	F (4, 80) = 0.4468	0.7744
		Spike Variant	F (4, 80) = 12.42	< 0.0001
		ACE2 Variant	F (1, 80) = 5.124	0.0263

S2 Table: N-glycans identified on ACE2-Fc and ACE2(NN)-Fc

Glycan Mass	Glycan Structure	Nomenclature	ACE2-Fc		ACE2(NN)-Fc	
			Average	S.D.	Average	SD
1851.96	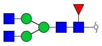	FA2	21.19%	0.021	21.04%	0.038
2056.06	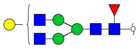	FA2G1	7.30%	0.004	9.53%	0.005
2097.08	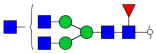	FA3	4.61%	0.003	6.58%	0.036
2243.14	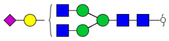	A2G1S1	1.85%	0.003	2.50%	0.001
2260.16	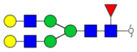	FA2G2	2.95%	0.005	3.42%	0.006
2342.21	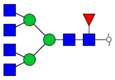	FA4	3.37%	0.000	2.79%	0.003
2417.23	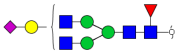	FA2G1S1	5.35%	0.000	5.14%	0.005
2447.24	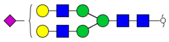	A2G2S1	2.76%	0.000	3.88%	0.004
2621.33	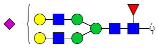	FA2G2S1	9.22%	0.000	5.61%	0.005
2662.36	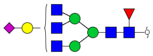	FA3G2S1	2.08%	0.002	2.71%	0.005
2808.41	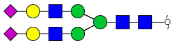	A2G2S2	1.95%	0.000	2.65%	0.001
2866.46	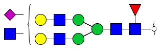	FA3G2S1	0.45%	0.006	1.36%	0.001
2907.48	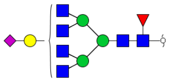	FA4G1S1	2.32%	0.000	1.52%	0.003
2982.50	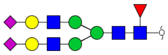	FA2G2S2	17.52%	0.009	9.54%	0.014
3070.56	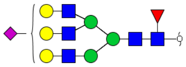	FA3G3S1	1.05%	0.001	1.52%	0.005
3227.63	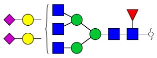	FA3G2S2	1.59%	0.000	2.66%	0.004
3431.73	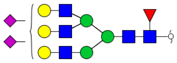	FA3G3S2	2.83%	0.002	2.97%	0.008

3472.76	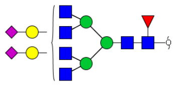	FA4G2S2	1.42%	0.001	1.70%	0.004
3676.86	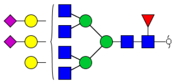	FA4G3S2	0.75%	0.001	0.84%	0.000
3792.77	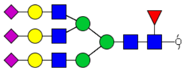	FA3G3S3	3.08%	0.004	2.61%	0.012
3880.90	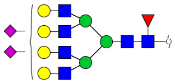	FA4G4S2	1.25%	0.001	3.54%	0.038
4038.03	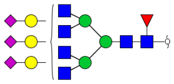	FA4G3S3	1.00%	0.002	1.63%	0.010
4242.128	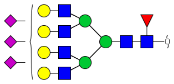	FA4G4S3	2.20%	0.004	2.74%	0.015
4603.30	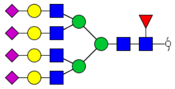	FA4G4S4	1.90%	0.004	1.59%	0.010

S.D. = Standard Deviation Yellow circles = Gal, Blue Square = GlcNAc, Red Triangle = fucose, purple diamond = Neu5Ac.

Supplementary figure

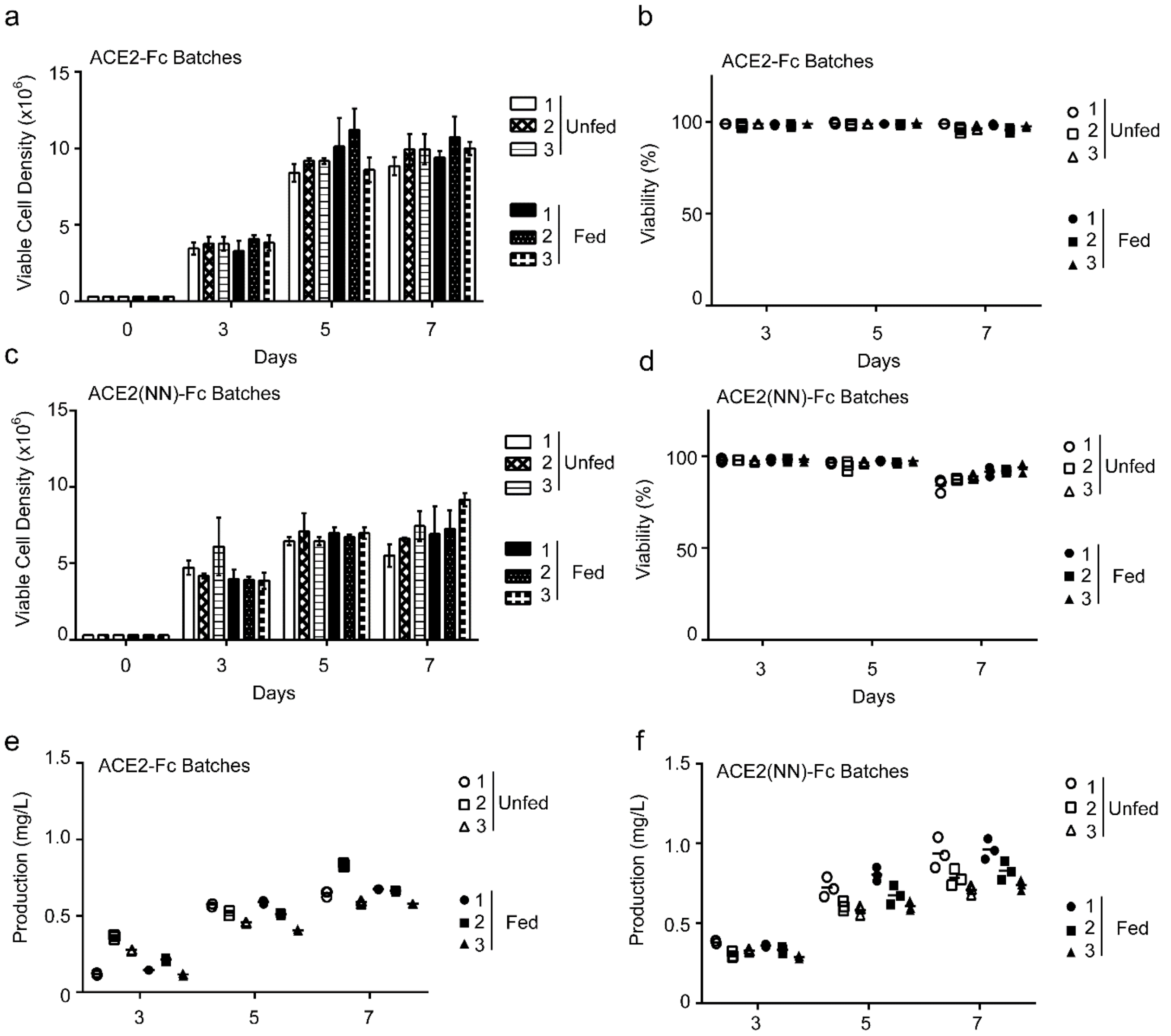

S1 Fig: Batch analysis of upstream manufacturing in ACE2-Fc and ACE2(NN)-Fc lines with fed and unfed conditions. Three separate batches with unfed and fed conditions were analyzed on days 3, 5, and 7 for (**a**) viable cell density (**b**) percentage viability (**c**) protein production.
